# Dung beetles as samplers of mammals in Malaysian Borneo—a test of high throughput metabarcoding of iDNA

**DOI:** 10.7717/peerj.11897

**Published:** 2021-08-13

**Authors:** Rosie Drinkwater, Joseph Williamson, Elizabeth L. Clare, Arthur Y.C. Chung, Stephen J. Rossiter, Eleanor Slade

**Affiliations:** 1 School of Biological and Chemical Sciences, Queen Mary University of London, London, United Kingdom; 2 Sabah Forestry Department, Forest Research Centre, Sandakan, Malaysia; 3 Asian School of the Environment, Nanyang Technological University, Singapore City, Singapore; 4 Department of Zoology, University of Oxford, Oxford, United Kingdom

**Keywords:** Biodiversity surveys, Southeast Asia, Scarabaeoidea, Invertebrate-derived DNA, Vertebrates, Tropical forests

## Abstract

Invertebrate-derived DNA (iDNA) sampling in biodiversity surveys is becoming increasingly widespread, with most terrestrial studies relying on DNA derived from the gut contents of blood-feeding invertebrates, such as leeches and mosquitoes. Dung beetles (superfamily Scarabaeoidea) primarily feed on the faecal matter of terrestrial vertebrates and offer several potential benefits over blood-feeding invertebrates as samplers of vertebrate DNA. Importantly, these beetles can be easily captured in large numbers using simple, inexpensive baited traps, are globally distributed, and occur in a wide range of habitats. To build on the few existing studies demonstrating the potential of dung beetles as sources of mammalian DNA, we subjected the large-bodied, Bornean dung beetle (*Catharsius renaudpauliani*) to a controlled feeding experiment. We analysed DNA from gut contents at different times after feeding using qPCR techniques. Here, we first describe the window of DNA persistence within a dung beetle digestive tract. We found that the ability to successfully amplify cattle DNA decayed over relatively short time periods, with DNA copy number decreasing by two orders of magnitude in just 6 h. In addition, we sampled communities of dung beetles from a lowland tropical rainforest in Sabah, Malaysia, in order to test whether it is possible to identify vertebrate sequences from dung beetle iDNA. We sequenced both the gut contents from large dung beetle species, as well as whole communities of smaller beetles. We successfully identified six mammalian species from our samples, including the bearded pig (*Sus barbatus*) and the sambar deer (*Rusa unicolor*)—both vulnerable species on the IUCN red list. Our results represent the first use of dung beetle iDNA to sample Southeast Asian vertebrate fauna, and highlight the potential for dung beetle iDNA to be used in future biodiversity monitoring surveys.

## Introduction

The development and application of molecular techniques to sequence DNA contained within environmental samples (eDNA), including water, soil, and air, has provided new opportunities for assaying biodiversity (Robson et al., 2016; Van der Heyde et al., 2020; Clare et al., 2021; Nguyen et al., 2020). In terrestrial systems, recent DNA-based studies have assayed vertebrate biodiversity by sequencing dietary DNA contained within the meals of invertebrates (iDNA), including leeches (Drinkwater et al., 2018; Tilker et al., 2020), carrion flies (Rodgers et al., 2017), and sandflies and mosquitoes (Kocher et al., 2017).

In the tropical forests of Southeast Asia, numerous studies have demonstrated the potential of using terrestrial leech iDNA for surveying biodiversity (Schnell et al., 2018; Tessler et al., 2018; Abrams et al., 2019; Fahmy et al., 2019; Drinkwater et al., 2021; Ji et al., 2020). The popularity of using leeches to sample vertebrates may stem from their abundance in the region, and their attraction to the human collector. Terrestrial leeches, however, have critical limitations in their use as biodiversity monitors. First, they are difficult to trap in a standardised way, with most leech iDNA studies using opportunistic collection methods (*e.g*., Schnell et al., 2018) or, more recently, hand-searching within fixed areas (*e.g*., Abrams et al., 2019; Drinkwater et al., 2021). Secondly, the group of blood-feeding terrestrial leeches with the potential for use in iDNA studies are geographically constrained to Southeast Asia, India, Australia, and Madagascar (Borda & Siddall, 2010; Schnell et al., 2018), and within these regions their occurrence is linked to humid forest habitats (Drinkwater et al., 2019).

As potential samplers of vertebrate DNA, dung beetles (superfamily Scarabaeoidea) offer several advantages over blood-feeders. First, they are a diverse and wide-ranging group that feed primarily on the faecal matter of terrestrial vertebrates and occupy most terrestrial habitats, with a global distribution ranging from temperate zones to the equatorial tropics (Nichols & Gardner, 2011). Second, dung beetles can be easily captured in large numbers using low-cost home-made traps, allowing for standardised sampling regimes (Nichols & Gardner, 2011). Indeed, in the Brazilian Amazon, Gardner et al. (2008) found that dung beetles are one of the most cost-effective taxa for biological surveys, while also having one of the highest ecological indicator values of the study taxa. Many species of dung beetle are considered generalists in their feeding preferences (Frank et al., 2018), allowing the detection of mammalian communities within an area even when only a few species of dung beetle are present. However, some dung beetle species are known to specialise on the dung of certain mammal taxa or feeding guilds (*e.g*., Raine & Slade, 2019), potentially making it possible to target specific vertebrate groups of interest. These feeding behaviours may allow the fine-scale tuning of iDNA studies, which could be beneficial for research with limited time or financial resources.

Although dung beetles may, in theory, present a promising new source of iDNA for biodiversity monitoring, to date there have only been three studies assessing their efficacy as samplers of mammalian DNA. Gómez & Kolokotronis (2016) used Sanger sequencing to recover DNA from the guts of individual dung beetles feeding on horse manure. Taking a similar approach, Gillett et al. (2016) applied shotgun sequencing to the guts of ten African dung beetles, and assembled a near-complete wildebeest mitogenome. More recently, Kerley et al. (2018) successfully applied metabarcoding to retrieve wild mammal DNA from the faeces of individual dung beetles. The duration of time over which mammal DNA can be retrieved after a feeding event is likely to be a key parameter in interpreting the results of any iDNA-based biodiversity assessments but has never been quantified for dung beetles. A long window of DNA persistence will increase the chances of retrieving amplifiable DNA in a randomly caught sample of beetles. Alternatively, although the chance of detectable DNA would be lower, a short window of DNA persistence would result in more accurate spatiotemporal information on vertebrate populations.

Here we aim to further assess the potential use of dung beetles as iDNA samplers for biodiversity studies. To this end, we ascertain, for the first time, the window of mammal iDNA persistence in the dung beetle gut. For this, we performed a controlled feeding experiment, focusing on a large-bodied species (*Catharsius renaudpauliani*) which occurs across Borneo. Additionally, we applied a metabarcoding protocol to pooled samples of small dung beetles, and gut contents of large-bodied dung beetles, to assess whether these multi-species assemblages can be used to assay wild mammal diversity.

## Methods

### Controlled feeding experiment

#### Sample collection and gut dissection

To measure the window of detection of mammal DNA within dung beetle guts, we conducted a controlled feeding experiment using individuals of the largest dung beetle species commonly occurring in our study area (*Catharsius renaudpauliani*). We collected 60 *C. renaudpauliani* individuals using standard human (*Homo sapiens*) dung baited live pitfall traps, which were deployed for 24 h at multiple locations as part of another study (see Parrett et al. (2019) for collection details). Individuals were maintained in sex-specific holding boxes, with moist sand and *ad libitum* cow dung for three days. Cow dung was used as it represented the only non-human dung that could be obtained in bulk from the surrounding area.

To measure the persistence of cow DNA in dung beetle guts, individual *C. renaudpauliani* were transferred to clean enclosures and starved for 48 h to purge any previously ingested dung from their systems. As very little is known about metabolism and digestion in this species, the purging time was based on a study by Upadhyay (1983) who found that full digestion occurred within 48 h in a congeneric species (*C. molossus*). Twenty grams of cow dung was then introduced into the boxes for approximately 1 h, allowing all individuals the opportunity to feed. Any remaining dung was then removed, and the enclosures were cleaned thoroughly. At 10 time points (0, 1, 2, 4, 6, 9, 12, 24, 48, 56 h post-feeding) six individual beetles (three females and three males) were selected *ad hoc* and frozen at −20 °C for an hour before decapitation. As rapid digestion of the dung was assumed, we maximised the number of early time points sampled post-feeding to capture patterns of DNA degradation. Once frozen, we removed the elytra and wings to reveal the gut within the abdominal cavity which is easily removed with forceps in this large species. Care was taken to only sample gut tissue, which was subsequently placed in a volume of RNALater 3–4 times greater than the tissue and stored in a −20 °C freezer for DNA preservation.

#### Quantification of DNA

DNA was extracted from all beetle guts following the protocol used in Drinkwater et al. (2018) for the extraction of iDNA from terrestrial leeches. This involved digesting each sample overnight in approximately five ml of a lysis buffer consisting of 10 mM Tris-HCl pH 8.0, 10 mM Sodium Chloride, 2% w/v Sodium Dodecyl Sulfate, 5 mM Calcium Chloride, 2.5 mM EDTA (pH 8.0), 40 mM Dithoithreitol, and 1% proteinase K solution. After lysis, DNA was extracted using a QiaQuick purification kit (Qiagen, Germantown, MD, USA) following the manufacturer’s protocol, but using reduced centrifuged speeds of 6,000 g. This protocol of homemade digestion buffer with DNA purification is a more cost-effective way of extracting pools of samples using greater volumes of buffer compared to Qiagen DNA extraction kits (see Drinkwater et al., 2018 and Schnell et al., 2018). Quantitative PCR (qPCR) was used to determine the concentration of cow DNA detected from the beetle guts at the experimental time points. The six DNA extracts from each time point were initially diluted by a factor of five to reduce PCR inhibition, following optimal practices for iDNA from previous work (Schnell et al., 2012; Bohmann et al., 2018). We amplified mammal DNA from the gut samples using the same 16s rRNA primers used in previous iDNA studies (Drinkwater et al., 2018; Schnell et al., 2018; Axtner et al., 2019) which target small (~95 bp) fragments of mammal DNA (Taylor, 1996). qPCR reactions were then set up in a total volume of 20 μl using SYBR green fluorescence as the marker and ran in triplicate. Each reaction consisted of 10 μl of SensiFAST mastermix (Bioline, UK), 0.8 μl of 10 μM primers, both forward and reverse, 7.4 μl of ddH20, with 1 μl of unknown DNA template. For quantification, we used a standard curve of eight samples of known DNA concentrations which were included in the qPCR plate alongside the unknown gut samples (standard curve in Fig. S1). For confirmation of the identity of the qPCR product, a subset of the reactions were sequenced using Sanger sequencing, and the species identified from the NCBI GenBank database with BLAST. The copy number of samples was determined from each qPCR cycle threshold (CT), and the intercept and slope of the standard curve using the equation:

copy number = 10^(CT – intercept)/slope^

The mean DNA copy number of each technical replicate per sample (*n* = 3) was calculated. The effect of time post-feeding on the number of DNA copies recovered was tested using a log-log linear regression model, with log_10_ DNA copy number as the response variable, and log_10_ time in hours (post-feeding) as the main effect.

### Sequencing of iDNA from multi-species communities

#### Sample collection and extractions

We set live pitfall traps, baited with human dung, in an area of continuous logged forest for 24 h (see Parrett et al. (2019) for methods). From these traps we collected either just individuals of the two largest bodied dung beetle species (*Catharsius renaudpauliani* and *C. dayacus*), or the whole community of smaller dung beetles (with any larger *Catharsius* spp. removed). *Catharsius* individuals were either dissected in the field or at Queen Mary University of London (dissection described above) and were stored in 3–4 times the gut (or body) volume of RNALater. Individual gut samples underwent tissue lysis, before extraction pools were constructed by combining 100 μl from each sample following the pooling protocol in Drinkwater et al. (2018). DNA extractions were conducted on these pools (see qPCR DNA extractions) except for one sample which was extracted as a single gut. For the smaller dung beetles, the total contents of two traps were stored in ethanol. Before DNA extraction, excess ethanol was allowed to evaporate and the trap contents were cleaned in freshwater. These samples were then mechanically crushed, without prior gut dissection, and split into three or four samples (samples shown in Table 1) with DNA extracted as above (see qPCR DNA extractions).

**Table 1:** Summary of samples used, including non-human mammal detections and proportion of human reads. Breakdown of samples used in this study along, which consisted of either a gut sample or a community trap sample. The number of (non-human) mammals detected in each sample is given along with the % of human reads per sample and % of target (non-human) mammal reads. These percentages are calculated on the filtered and normalised read counts.

Sample	Type	Sample content	Mammal detections	% Target mammal reads	% Human reads
1	Gut	Pool (6 individuals)	0	0.0	94.0
2	Gut	Pool (6 individuals)	0	0.0	100.0
3	Gut	Pool (6 individuals)	4	38.3	61.7
4	Gut	Pool (6 individuals)	0	0.0	100.0
5	Gut	Single	2	71.9	28.1
6	Community	Trap 1 subsample A	0	0.0	100.0
7	Community	Trap 1 subsample B	2	41.3	51.7
8	Community	Trap 1 subsample C	1	0.05	97.6
9	Community	Trap 1 subsample D	0	0.0	20.6
10	Community	Trap 2 subsample A	0	0.0	100.0
11	Community	Trap 2 subsample B	3	75.2	24.8
12	Community	Trap 2 subsample C	0	0.0	100.0

#### PCR amplification and sequencing

We used the same primers targeting mammalian 16S rRNA as in the controlled-feeding experiment (Taylor, 1996) and followed the same laboratory protocols for sequencing of leech iDNA in Drinkwater et al. (2018). First, each pooled or individual DNA extract was amplified using uniquely tagged 16 s primers (Binladen et al., 2007), with extraction blanks and negative PCR controls included in each PCR run. The reactions consisted of 1 μl of template DNA in 0.2 mM of 10×buffer, 2.5 mM MgCl2, 1 unit DNA polymerase (AmpliTaq Gold; Applied Biosystems, Foster City, CA, USA), 0.2mM dNTP mix (Invitrogen, Carlsbad, CA, USA), 0.5 mg/ml BSA, and 0.6 μM of the forward and reverse primer to make a final reaction volume of 2 μl. We used thermocycling conditions of 95 °C for 5 min, then 40 cycles of 95 °C for 12 s, 59 °C for 30 s and 70 °C for 20 s, with a final extension time of 7 min at 70 °C. Amplification was checked on a 1% agarose gel and successful reactions were combined for DNA amplicon libraries (Carøe et al., 2017). For efficiency, the samples were combined into DNA libraries based on approximately equimolar concentrations calculated on band strength, (~10 μl), along with leech iDNA samples from another study, which had been treated in the same way (Drinkwater et al., 2018). Each DNA library consisted of approximately 10 amplicon pools (mixtures of leech and dung beetle amplicons) with no repeated tags and at least two base pair mismatches per tag so that samples could be identified even if sequencing error or tag jumping occurred (Schnell, Bohmann & Gilbert, 2015). DNA amplicon libraries were then sequenced in two runs on an Illumina MiSeq using V2 chemistry 2 × 150 bp at The Genome Centre, Queen Mary University of London.

#### Bioinformatics and taxonomic identification

We merged forward and reverse reads with AdapterRemoval version 2 (Schubert, Lindgreen & Orlando, 2016) and sorted samples by their unique 16S primer tags, allowing the identification of the original sample before filtering using DAMe (Zepeda-Mendoza et al., 2016; following version updates at https://github.com/shyamsg/DAMe). Unpaired reads were removed and remaining reads were filtered using a minimum length cut-off of 90 bp, and a restrictive PCR filtering approach where only sequences found in at least two out of three PCRs are retained (Alberdi et al., 2018). We clustered reads into operational taxonomic units (OTUs) at 97% similarity using SUMACLUST (Mercier et al., 2013) and OTU copy numbers were normalised. OTUs were then checked for chimeras using mothur (Schloss et al., 2009) and further filtering of OTUs was conducted using LULU (Frøslev et al., 2017). OTUs were identified using a BLAST search against a customised reference database, resulting in a list of taxa for each dung beetle gut iDNA sample. The reference database contained all available 16S mammal sequences for Bornean mammals and known lab contaminants (Table S1). Where reference sequences did not exist for a species, a closely related taxon was included. Due to our small sample size and the exploratory nature of the study, we present the results as descriptive data.

## Results

### Window of DNA persistence in *C. renaudpauliani* guts

**Figure 1:**
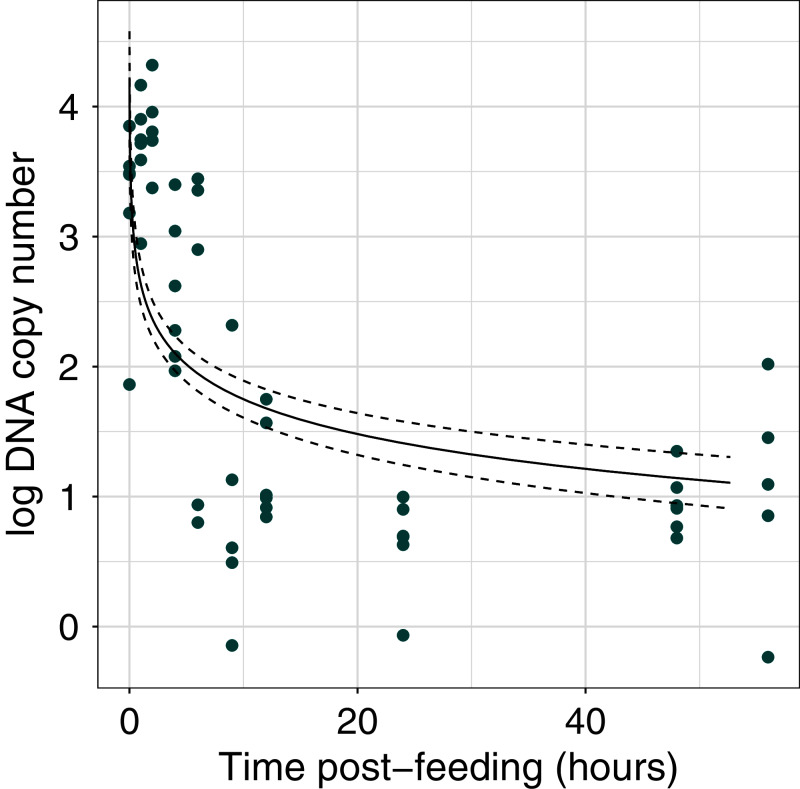
Dung beetle iDNA decrease with time since experimental feeding. Comparison of log DNA copy number as a function of time post-experimental feeding. DNA copy number calculated from qPCR experiment at fixed time points post-feeding. The points represent the mean value for each sample at a time point (*n* = 3 technical replicates).

The efficiency of the qPCR reactions (R^2^) was greater than 0.99 (Fig. S1). DNA copy number declined with time post-feeding (log-log regression, estimate = −0.891 ± 0.136, t = 11.86, *df* = 55, *p* < 0.001; Fig. 1, Fig. S2) By 6 h post-feeding DNA copy number showed a sharp decrease of two orders of magnitude (Fig. 1).

### Sequencing summary from multi-species communities

From the two paired end sequencing runs we retrieved a total of 26,623,610 reads (run one = 12,229,874 and run two = 14,393,736), which was on average was 1,111,806 reads per library for run one (number of libraries = 11) and 654,260 reads per library for run two (number of libraries = 22). The paired read merging rate was between 84% and 97% success across all libraries and runs. After removing adapters, filtering and only retaining those reads present in two out of the three PCR replicates 7,171,305 reads remained (run one = 3,321,805 and run two = 3,849,500). After sorting by unique amplicon tag this resulted in a final set of 327,482 reads assigned to dung beetle samples (from run one = 34,445 and run two = 293,037) (see Table S1 for breakdown of filtered reads per dung beetle sample). This was on average 29,771 reads per dung beetle sample (11 samples).

### Identification of mammal species from dung beetle assemblages

We recovered 12 detections of six non-human mammalian taxa from both sample types, based on guts and communities. These mammals were from five families and represented some of the common species in the area, including bearded pig (Suidae: *Sus barbatus*), sambar deer (Cervidae: *Rusa unicolor*) and mousedeer (Tragulidae: *Tragulus* sp.) (Table 2). Five OTUs were taxonomically assigned using BLAST, with a percent identity >99% (human, bearded pig, sambar deer, mousedeer, and banded civet (Viverridae: *Hemigalus derbyanus*)), while two OTUs had a lower percent identity match of 91% (muntjac (Cervidae: *Muntiacus* sp.) and porcupine (Hystricidae: *Hystrix* sp.) (Table S2). All traps recovered high levels of human DNA contamination (Table 1).

**Table 2:** Mammal detections in dung beetle iDNA. Bornean mammal taxa detected in dung beetle iDNA. The total number of samples the taxa was found in is given, along with a breakdown of the detections within each sample type in brackets, either gut samples or trap samples. The post-filtering read count is also provided.

Common name	Family	Taxa assigned	Detections (gut, trap)	Normalised read count
Bearded pig	Suidae	*Sus barbatus*	3 (2, 1)	14,861
Sambar deer	Cervidae	*Rusa unicolor*	3 (1, 2)	23,455
Muntjac	Cervidae	*Muntiacus* sp	1 (0, 1)	36,744
Mousedeer	Tragulidae	*Tragulus* sp	3 (2, 1)	36,902
Porcupine	Hystricidae	*Hystrix* sp	1 (1, 0)	1,214
Banded civet	Viverridae	*Hemigalus derbyanus*	1 (0, 1)	20

## Discussion

We have demonstrated that iDNA from mammalian sources can be recovered from the digestive tracts of tropical forest dung beetles using a high throughput sequencing pipeline developed for leech-based biodiversity surveys (Drinkwater et al., 2018). Using a controlled feeding experiment, we found that there was rapid digestion and fast passage of cow dung through the beetle gut, with a two orders of magnitude decrease in DNA copy number just 6 h post-feeding. In addition, we were able to sample mammalian DNA from both the gut contents of large dung beetles, and from the sequencing of entire communities of smaller beetles.

There has been very little previous work on digestion rates in dung beetles, but our findings broadly corroborate those of Upadhyay (1983), who reported a short digestion window of 48 h in (*Catharsius molossus*), a congener of our study species. This is in marked contrast to the blood feeding medicinal leech (*Hirudo medicinalis*) for which Schnell et al. (2012) found that iDNA could be detected for up to four months after a feeding event. The clear difference in the time window of detection offered by dung beetles and leeches highlights the potential benefit of combining these two invertebrate samplers to target mammal diversity. At the same time, however, this is a preliminary experiment conducted under field conditions in Borneo, in which cow dung was used for both the experimental and pre-experimental feeding. For this reason, we cannot rule out the possibility that cow DNA detected post-feeding could have persisted from a previous feeding event. However, we detected very little cow DNA ~20 h post-feeding and the beetles were given a 48-h purging window before any experimental work was carried out.

Our assays of multi-species beetle communities led to the detection of six mammalian (non-human) taxa, representing five taxonomic families. Three taxa were resolved to species level, whereas three could only be confidently identified to genus, as there are two congeneric species present across the site. The most frequently detected mammals were the locally common and larger-bodied species (bearded pig, muntjac and sambar deer) indicating these ungulate species may be a key dietary resource for dung beetles. In addition, porcupine (*Hystrix*. sp) was also positively identified and could be assigned to one of two *Hystrix* species in Borneo, the endemic thick-spined porcupine or the Malay porcupine, both of which are relatively large and abundant. We also recorded low levels of DNA from the banded civet from one sample, which is a species of conservation concern due to declining population trends, and is listed as near threatened on the IUCN red list (Ross et al., 2015).

The substantial number of detections from only 12 samples suggest that this method holds promise as a complementary method to camera trapping and scat surveys in tropical forests. These traditional vertebrate sampling techniques are time- and cost-intensive in tropical forest conditions, with scat surveys in particular difficult due to high dung turnover rates by beetles (Norris & Michalski, 2010). Thus, the speed, standardisation, and cost-effectiveness of sampling using dung beetles (see Gardner et al., 2008), means that it could be beneficial to use dung beetle iDNA surveys alongside standard methods to supplement detection data. The validation of iDNA surveys compared to camera trapping is an active area of research (Rodgers et al., 2017; Gogarten et al., 2019; Hanya et al., 2019). Studies have shown that by combining the results of iDNA with camera traps, researchers can increase the confidence of site occupancy probability estimates, therefore making results more relevant to wildlife monitoring programmes (Abrams et al., 2019).

We note that a high amount of human DNA was recovered even when using the most sterile techniques, and our results indicate low non-human mammal detections in samples where human DNA proportions are highest. Although some of this DNA will have arisen through laboratory or field contamination, it is likely that it may also represent true feeding events. Our study was conducted in a modified landscape consisting of logged forest and oil palm agriculture, with associated human settlements and industrial infrastructure alongside a research field station. Humans could therefore represent an abundant and consistent food source for the dung beetles in this area. The use of human blocking primers might therefore be advantageous in future dung beetle iDNA studies. By reducing the amount of human DNA amplified, such blocking primers would free up sequencing depth for potentially rare DNA detections, a technique that has been used successfully in ancient genetic and iDNA studies (Boessenkool et al., 2012; Rodgers et al., 2017)

The possible temporal threshold of DNA persistence in the guts of *C. renaudpauliani*, suggests that when mammal DNA is detected, feeding is likely to have occurred within days of being trapped. Dung beetles are attracted to fresh dung, which is removed quickly in tropical forests (with even large dung piles completely removed within 24 h, Slade, Mann & Lewis, 2011). Our findings therefore suggest that the mammals detected by iDNA occupied the area within the temporal window of the trapping campaign. Although this requires further validation, the potential to “time-stamp” iDNA detections in this way could be beneficial for conservation applications, presenting a promising benefit over current leech-based studies. Our proof-of concept study clearly highlights the usefulness of combining multiple iDNA samplers, offering the potential of targeting two different windows of detection, one short term (*i.e*., beetles) and one longer term (*i.e*., leeches).

### Permits

Access and export permits to RD and EMS—JKM/MBS.1000-2/2 (34) JKM/MBS.1000-2/3 JLD.2 (107) and JKM/MBS.1000-2/3 JLD.3 (44).

## Supplemental Information

10.7717/peerj.11897/supp-1 Supplemental Information 1 qPCR standard curve showning an efficiency of 99%. In black are the CT values of each standard of known concentration. Using the slope of the line (where slope = −3.3 and intercept = 35.72) the efficiency of the reaction is calculated as 99.76% using the standard equation E = −1+10^(−1/slope)^. For qPCR, the desired range of efficiency is between 90–110%.Yellow points refer to the mean CT values for the gut samples at each time point post feeding.

10.7717/peerj.11897/supp-2 Supplemental Information 2 Raw values of DNA copy number for each sample at each time point post-feeding. Green points show the raw DNA copy number and black diamonds are the mean value for each time point.

10.7717/peerj.11897/supp-3 Supplemental Information 3 Filtered read counts per PCR replicate. Filtered read counts per PCR replicate for each dung beetle sample. Samples were either a pooled extraction of guts, a single gut extraction or trap community (3 extractions from each of the two traps). Read counts are after merging with AdapterRemoval and sorting unique amplicon tags and filtering with the DAMe pipeline.

10.7717/peerj.11897/supp-4 Supplemental Information 4 OTU summary table. Two tables (A) OTU by sample matrix and (B) taxonomic assignments with blast scores from LULU.

10.7717/peerj.11897/supp-5 Supplemental Information 5 16s Bornean mammal reference database list. Taxa included in the 16S reference database. Modified from Drinkwater et al., 2018, as the same reference database was used for these analyses. All publicly available sequences from GenBank.

10.7717/peerj.11897/supp-6 Supplemental Information 6 The results of the experimental feeding qPCR, with the CT values for the standards and the experimental samples.

## Additional Information and Declarations

### DNA Deposition

The following information was supplied regarding the deposition of DNA sequences: The genetic sequencing data is available on the NCBI Short Read Archive: PRJNA672059.

### Data Availability

The following information was supplied regarding data availability: The raw qPCR data for the feeding experiment, the filtered read numbers and the OTU sequences and hit table for the unknown dung beetle guts are available in the Supplementary Files.
