# Supplementary figures and images for: Dung beetles as samplers of mammals in Malaysian Borneo—a test of high throughput metabarcoding of iDNA
Source: PeerJ. 2021 Aug 13;9:e11897. doi: 10.7717/peerj.11897 (PMC8366524; doi:10.7717/peerj.11897)

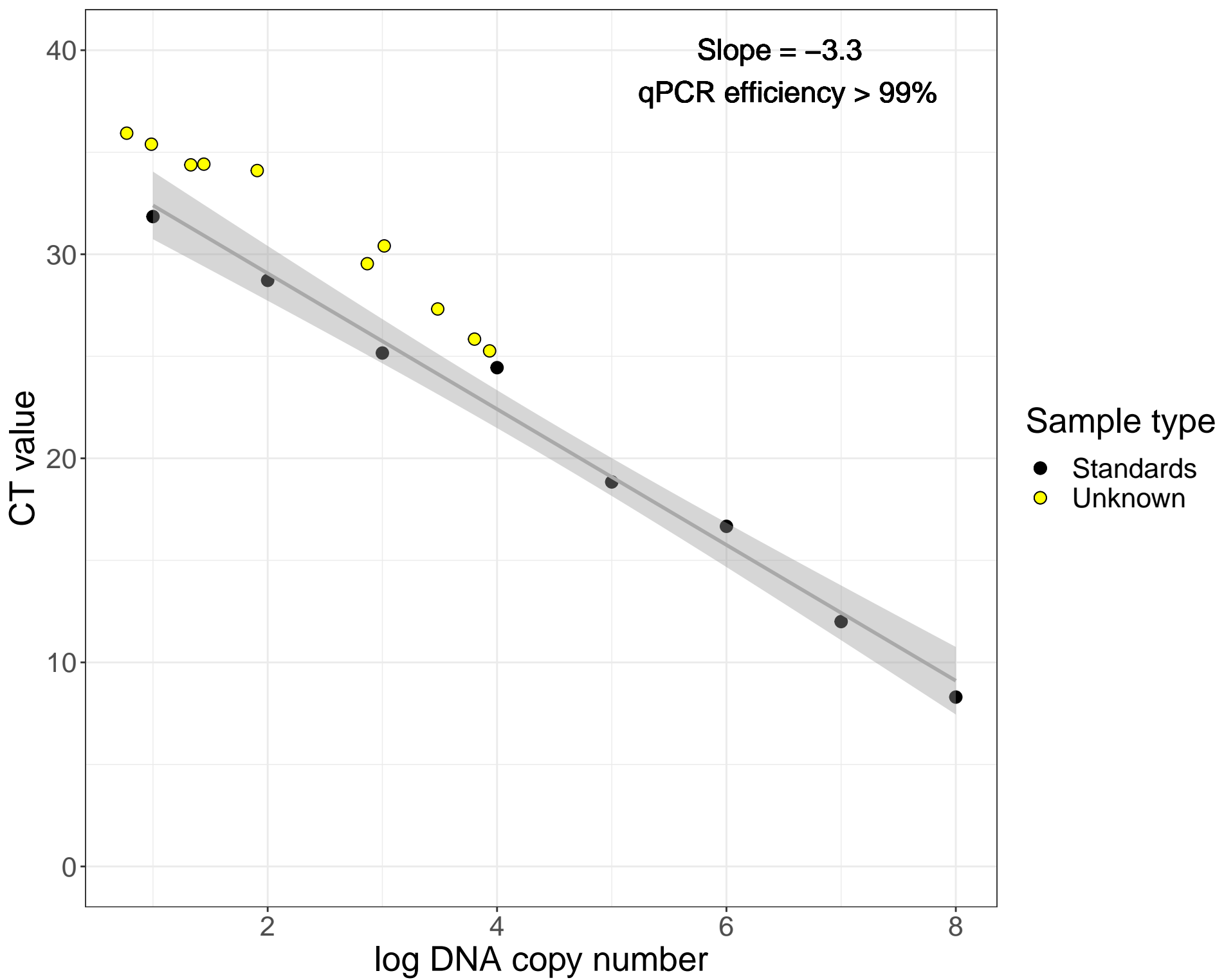

Supplement: Supplemental Information 1 — In black are the CT values of each standard of known concentration. Using the slope of the line (where slope = −3.3 and intercept = 35.72) the efficiency of the reaction is calculated as 99.76% using the standard equation E = −1+10(−1/slope). For qPCR, the desired range of efficiency is between 90–110%.Yellow points refer to the mean CT values for the gut samples at each time point post feeding. [file peerj-09-11897-s001.pdf]

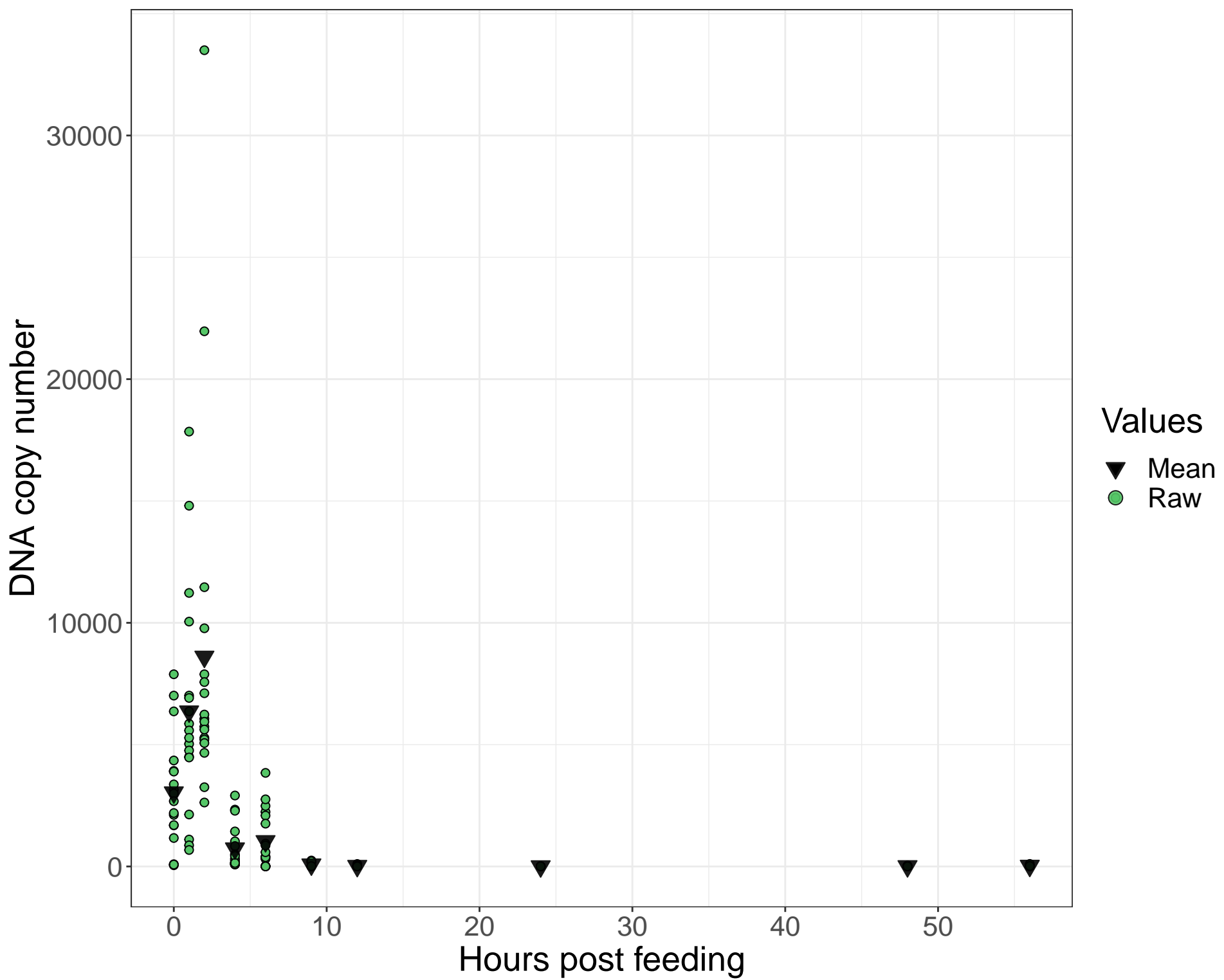

Supplement: Supplemental Information 2 — Green points show the raw DNA copy number and black diamonds are the mean value for each time point. [file peerj-09-11897-s002.pdf]
